# Resistivity method-based rock core orientation experimental protocol

**DOI:** 10.1371/journal.pone.0342912

**Published:** 2026-03-02

**Authors:** Jiahuan He, Zhijuan Tang, Qiang Kang, Jiaqi Wang, Li Wang, Hongyu Yao, Qingxiu Zhang, Xingwang Tian, Hongbin Chen, Linling Zhang

**Affiliations:** 1 Exploration and Development Research Institute, Southwest Oil and Gas Field Company, PetroChina, Chengdu, Sichuan, China; 2 Department of Engineering Mechanics, Tsinghua University, Beijing, China; 3 Petrophysics key laboratory, Southwest Oil and Gas Field Company, PetroChina, Chengdu, Sichuan, China; Henan Polytechnic University, CHINA

## Abstract

Restoring core orientation is critical for subsurface characterization but remains challenging as over 99% of cores are obtained through conventional drilling. We propose a novel, non-destructive method based on discrete radial resistivity measurements of full-diameter cylindrical cores. Unlike image-matching techniques, our approach directly correlates azimuthal resistivity values with downhole electrical imaging logs, eliminating the need for specialized software. This method reduces measurement time to less than 8 hours per sample and increases throughput to ~150 samples per operator annually, representing a 3–3.75-fold efficiency gain. Applied to Well ST12, it achieved an orientation accuracy of ±15° in anisotropic reservoirs. Measurement accuracy can also be improved as needed. The technique offers a cost-effective, scalable, and widely applicable solution for orienting conventionally acquired cores, enabling enhanced geological and geomechanical analysis.

## Introduction

Geological resources represent one of the Earth’s most abundant resource categories. Whether for conventional oil and gas or geothermal energy development as a renewable resource, obtaining geological information through physical core samples lays a solid foundation for more rational engineering design.Since over 99% of cores worldwide are acquired through conventional drilling coring [1–3], determining core orientation during the trip out of the hole remains challenging. Nevertheless, determining the original orientation of cores within the formation allows exploration professionals to accurately understand geological structures, sedimentary facies, reservoir permeability, and paleocurrent directions [4–7]. It also provides critical evidence for fracturing engineers to determine in-situ stress field directions [8–10]. The global engineering geology community has consistently prioritized core orientation research, regarding it as a key exploration technology [11]. Accurate, economical, and efficient restoration of in-situ core orientation is highly important in many fields [12–15]. These include solid mineral drilling and mining, oil and gas exploration and development, continental and oceanic scientific research, geological hazard prediction, and geotechnical engineering construction.

To address core orientation, engineers [16,17] have primarily employed two approaches: pre-design and post-analysis. The most direct.method is pre-design through directional coring [18,19]. This involves attaching directional devices to coring tools to mark the core, recording directional data of the primary marker while simultaneously measuring well inclination and azimuth [20]. The retrieved core bearing directional markings enables orientation restoration [21]. Its main advantage is straightforward principle and execution [22–25]. Numerous European and American countries have developed proprietary technologies following this approach, including the Craelius core orientation device, Odsers orienter, Christensen directional coring tool, the former Soviet Union’s K-5 core orienter, Sweden’s Atlas core orienter, Britain’s Archway core orienter, and Germany’s KTB core orientation system. Unfortunately, directional coring requires pre-job planning. Based on field crew experience, it carries significant operational risks, limiting its application to only a minority of wells to date.

Alternative methods have been studied for orienting cores from wells not predesigned for this purpose, with image matching and paleomagnetic analysis being the most common. Image matching [26–28] involves correlating borehole wall images with scanned core surface images to determine orientation. Its primary drawbacks are time-consuming core imaging and the need for specialized image processing software [29–31]. Rogers et al. (2000) [16] matched resistivity imaging with downhole electrical imaging logs to determine the clock orientation of cores in the formation. Their approach essentially provides only an image-based relative orientation, making it, strictly speaking, an image-matching method. In this study, we measure resistivity in different directions on full-diameter cores, obtaining discrete resistivity values, which are then compared with corresponding points from electrical imaging logs. The advantage of our method is that it requires measuring resistivity only in specific directions, taking less time than fully scanning cores to generate images, and it does not require specialized image-processing software, resulting in lower costs and higher efficiency. Following the Wenchuan Earthquake, Nie (2012) et al. [32] utilized ultrasonic imaging data integrated with core scan images for comprehensive processing and analysis. They first determined the core depth, and then compared core scan images with acoustic imaging log images to obtain the orientation of the cores. In 2013, Nie et al. [33] identified natural fractures, borehole breakouts, drilling-induced fractures, and drilling-enhanced natural fractures through core and imaging log analysis, and conducted statistical evaluations. Through two sets of conjugate fractures, they indicated the direction of the nearly vertical maximum paleo-principal stress, which likely formed as associated structures under the influence of the local stress field. Based on this, they inferred the average direction of the maximum horizontal principal stress in the target interval, which aligns with the stress state of the Yingxiu-Beichuan Fault Zone—one of the main seismogenic faults of the Wenchuan Earthquake.

Paleomagnetic analysis [34,35], another frequently used technique, requires cutting the core into cuboid samples and necessitates a magnetically shielded laboratory environment. By comparing the remnant magnetic signals on the core with the formation’s magnetic signature, orientation can be inferred. Beyond requiring specialized facilities, this method lacks universality for cylindrical samples and cannot achieve non-destructive testing. Cutting cores into cuboids inevitably damages these valuable physical samples, which represent irreplaceable data obtained at great cost from the subsurface. Additionally, wire-cutting of samples typically takes one working day, followed by marking and directional recording. The process usually requires a magnetically shielded laboratory environment to isolate the primary remanent magnetization. Preparation and testing for each sample analysis take 2–6 hours. After testing, demagnetization vector diagrams and intensity decay curves must be plotted. Specialized software is then used to identify stable magnetic components. Finally, the primary remanent magnetization direction and its geological significance are determined in combination with the geological context. This entire process may take from several days to weeks. Taking the relevant laboratory of Southwest Oil & Gas Field Company as an example, approximately 40–50 paleomagnetic experiments are conducted annually. Cutting cores into cuboid specimens, under current technological constraints, necessitates drilling new samples if geoscientists need to investigate physical properties beyond the three orthogonal directions of the cuboids, leading to substantial wastage of collected specimens. Cylindrical cores are mostly retrieved from underground formations thousands of meters deep, making sampling extremely costly.

Therefore, in the context of “carbon peak and carbon neutrality,” there is a need to develop a new core orientation method. This method should not require specialized coring procedures or laboratory environments. It should be applicable to readily available cylindrical samples through simplified, cost-effective processes that align with sustainable development principles. Such a method would demonstrate strong operational feasibility and practical value for widespread adoption.

Resistivity logging and electrical imaging logging are currently common logging methods in oil and gas exploration [36]. Resistivity parameters are widely used in the evaluation of geotechnical engineering parameters due to their cost-effectiveness. Akingboye (2023a) [37] jointly utilized seismic P-wave velocity (Vp), resistivity (ρ), and borehole-based RQD datasets to assess the geotechnical parameters of a typical tropical granite zone in southern Penang Island, Malaysia, significantly reducing the uncertainty and economic costs associated with borehole-based RQD evaluations in large-scale surveys. Akingboye (2023b) [38] systematically classified the soil–rock profile from the surface to the subsurface into residual soil, completely weathered rock, moderately weathered rock, and intact/fresh granite bedrock based on rock mass quality and condition by integrating borehole RQD models with SRT-RQD models to complement resistivity models. The study also identified fractures in various directions and with multiple axes. The advanced applications of electrical resistivity tomography (ERT) and seismic refraction tomography (SRT) are crucial for understanding dynamic processes from the surface to the shallow crust, providing key information on resistivity and velocity structures for geological and geohazard assessments. In recent years, machine learning (ML) techniques have been increasingly applied in these geophysical methods [39], particularly in the joint analysis of ERT and SRT, by optimizing nonlinear inversion processes and enhancing the interpretation of complex subsurface conditions. Even amid the widespread adoption of machine learning today, resistivity parameters continue to provide indispensable foundational data.

If these techniques can be utilized in combination with core analysis to achieve core orientation, it will hold significant potential for widespread application. To address this gap, we hypothesize that the discrete directional resistivity signature of a full-diameter core, quantitatively measured under in-situ saturation conditions, can be directly correlated with high-resolution electrical imaging logs to accurately restore its original orientation. This study aims to develop and validate a novel resistivity-based core orientation method, distinct from prior image-matching techniques. Our approach focuses on measuring and comparing discrete resistivity values (rather than full images) at targeted azimuths. This operational shift reduces core measurement time to less than 8 hours per sample and increases annual throughput to approximately 150 samples per operator—representing a 3 to 3.75-fold improvement in efficiency compared to conventional imaging methods, while eliminating reliance on specialized image-processing software. The method is inherently non-destructive, requiring no cutting of cylindrical cores and thereby preserving 100% of these invaluable geological samples. We further target an orientation accuracy of ±15° under standard 30° measurement intervals for anisotropic reservoirs. Measurement accuracy can also be improved as needed. By integrating routine logging data with a streamlined laboratory workflow, this technique offers a cost-effective, scalable, and practical solution for orienting the >99% of cores acquired through conventional drilling.

## Materials and methods

To achieve in-situ parameter measurements on core samples, it is essential to first determine the fluid saturation corresponding to the specific well depth. For high-maturity gas reservoirs, the water saturation at the target depth can be directly referenced. First, a full-bore-diameter core sample is fully saturated with formation water. Then, a gas-displacing-water process is employed to establish saturation conditions consistent with those in the formation. The full-bore-diameter core samples were first evacuated to a vacuum level of 6 × 10⁻² Pa. Under this vacuum, they were saturated with formation water for 4 hours. Subsequently, the formation fluid was pressurized to 30 MPa. After 48 hours of saturation under pressure, the samples reached a fully saturated state. This procedure follows the relevant steps of API RP 40 for fluid saturation, which is a common practice in the petroleum industry.

During the rock resistivity testing, the gas-displacement-water experiment was conducted in accordance with the SY/T 6712-2023 “*Specification for Laboratory Measurement of Electrical Parameters of Rock Samples*”. The method used is the ambient temperature and pressure gas-displacement gravimetric method for measuring resistivity under decreasing saturation. Common nitrogen or air served as the displacement gas, with a displacement pressure of 3 MPa. The confining pressure was maintained at 2 MPa above the displacement pressure. After removing surface moisture from the saturated core samples, the samples were weighed on an electronic balance. The water saturation at each stage was calculated by recording the mass change of the samples over different time intervals. The resistivity values of the rock samples in different directions were simultaneously recorded.

In parallel, the cylindrical sample radial resistivity measurement method [1,40,41] was used to test resistivity values in different directions. The key formulas for this radial resistivity testing method have been derived in detail by He et al [1,41] (2020).

The radial resistivity formula for rock core is given as follows


$$\rho =\frac{K}{K^{\prime }}LR,$$ (1)

where, *ρ* denotes resistivity, in Ω.m;


$$K\left(\alpha \right)=\int_{\text{0}}^{\text{1}}\frac{dt}{\sqrt{\left(1-t^{\text{2}}\right)\left(1-\alpha t^{\text{2}}\right)}},$$

In the formula, *K*′ is the derivative of *K*, where *K*′ is the complete elliptic integral of the first kind with modulus sin*α*, and *K* is the complete elliptic integral of the first kind with modulus cos*α*. For computational convenience, the curved electrode’s arc angle *β* was set to *π*/2. Hence, *G* = *K*′/*K* = 1. In that case, ρ = *LR*.

*K*’ denotes the derivative of *K*;

2*α* denotes the supplementary angle to the central angle of the curved electrode sheet contacting the core;

*L* denotes the core length, in m;

*R* denotes the apparent resistance value, in Ω.

Based on the radial resistivity measurement results, the rotation angles corresponding to the maximum and minimum resistivity values (*θ*_max_ for *R*_max_ and *θ*_min_ for *R*_min_) can be identified. These orientations are then marked on the core. Concurrently, by examining the darkest and brightest spots near the coring depth in the resistivity image logging data [37] and recording the corresponding formation azimuth, the core’s original in-situ orientation within the formation can be determined.

**Fig 1:**
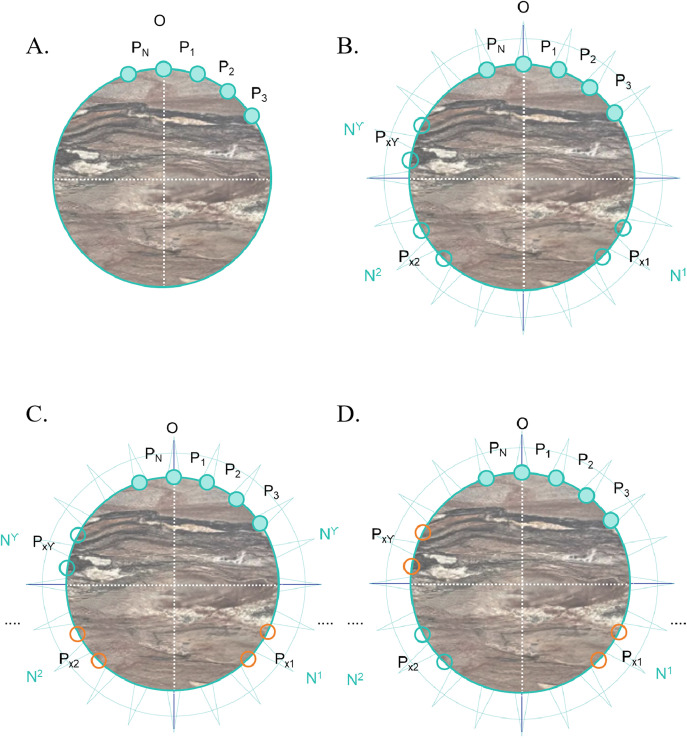
The process of marking directional resistivity for core samples. (A) This figure illustrates the first procure, which involves dividing the cylindrical rock core into equidistant segments and conducting resistivity tests on each segment. (B) This figure presents the second procure, in which positions with multiple maximum resistivity values are labeled. (C) This figure depicts the scenario where all data points fall within one complete cycle relative to the origin point “O”. (D) This figure explains the scenario in which multiple peak segments occur, with the latter segment just crossing the origin “O,” resulting in an “overlapping loop” condition.

In many cases, the brightest and darkest spots in the electrical image logging data are not distinct. To address this, the following procedure can be employed:

1. Designate a point on the core as reference point “O”. Divide the core circumference into N equal segments over 360°. Measure and record the resistivity value at each segment position. Label the positions sequentially as P_1,_ P₂, P₃,..., Pₙ, as shown in Fig 1A.
2. Identify the indices *N*₁, *N*₂,..., *N*ᵧ corresponding to the positions P₁ to Pₙ where the maximum resistivity values occur. Here, *γ* (*γ* ≤ N) is the number of maximum resistivity values. Define a set dn comprising values d_n1,2_, d_n2,3_,..., d_nγ,γ+1_,..., d_n2γ-1, 2γ_, as shown in Fig 1B.

Calculate the values in the dn set as follows:

For 1 ≤ i ≤ γ-1: dnᵢ,ᵢ + 1 = Nᵢ + 1 – Nᵢ, as shown in Fig 1C.

For i = γ: dnᵢ,ᵢ + 1 = dnᵢ,₁ = N₁ + N – Nᵢ, as shown in Fig 1D.

**Fig 2:**
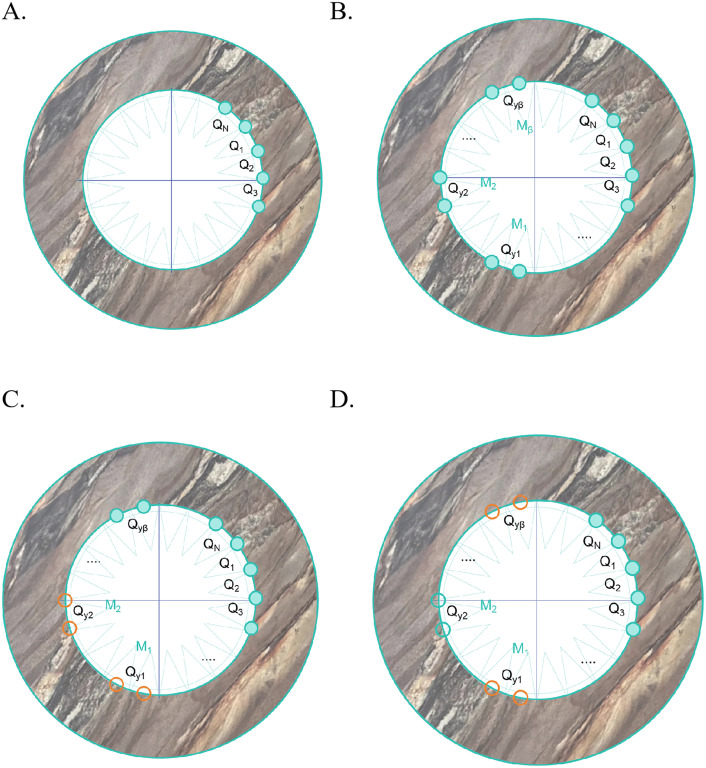
The process of marking directional resistivity for electrical imaging logging. (A) This figure illustrates the third procure, which involves dividing the resistivity image into equidistant segments and then calculating the respective resistivity values. (B) This figure explains the fourth step, labeling positions with multiple maximum resistivity values on the resistivity image. (C) This figure depicts the scenario on the resistivity image where all points fall within a single cycle. (D) This figure describes the situation on the resistivity image where two peak segments occur, with the latter segment resulting in an “overlapping loop” condition.

1. Divide the borehole circumference at the coring depth into N equal segments over 360°. Record the resistivity values from the resistivity image logging data for each segment position. Label these positions sequentially as Q₁, Q₂, Q₃,..., Qₙ, as shown in Fig 2A.
2. Identify the indices M₁, M₂,..., Mᵦ corresponding to the positions Q₁ to Qₙ where the maximum resistivity values occur. Here, β (β = γ) is the number of maximum resistivity values. Define a set dm comprising values dm₁,₂, dm₂,₃,..., dmᵦ,ᵦ + 1, as shown in Fig 2B. Calculate the values in the dm set as follows:

For 1 ≤ *i* ≤ *β*-1: dm_i,i+1_ = Mᵢ_+1_ – Mᵢ, as shown in Fig 2C.

For i = β: dm_ni,i+1_ = dm_ni,1_ = M_1_ + N - M_i_, as shown in Fig 2D.

1. Sequentially compare the d_n_ set values dn_1 +η,2 +η_, dn_2 +η,3 +η_,..., dn_y +η,y+1 +η_ with the d_m_ set values dm_1,2_, dm_2,3_,..., dmᵦ,_1_ for *η* = 0, 1, 2,..., *γ*-1.

When the dn set values match identically in sequence with the dm set values, record the current value of (1 + *η*) as *α*. Correlate the position information of P_n_^3^ with the position information of Q_M_^1^ to determine the core’s original formation orientation.

In specific implementation scenarios where *β* = *γ* = 3:

Set *η* = 0. Sequentially compare the values dn_1,2_, dn_2,3_, dn_3,4_ (i.e., dn_3,1_) with dm_1,2_, dm_2,3_, dm_3,4_ (i.e., dm_3,1_).

If dn_1,2_ ≠ dm_1,2_, or dn_2,3_ ≠ dm_2,3_, or dn_3,1_ ≠ dm_3,1_, then set η = 1. Sequentially compare the values dn_2,3_, dn_3,4_, dn_4,5_ (i.e., dn_2,3_, dn_3,1_, dn_1,2_) with dm_1,2_, dm_2,3_, dm_3,4_.If still no match, set η = 2. Sequentially compare the values dn_3,4_, dn_4,5_, dn_5,6_ (i.e., dn_3,1_, dn_1,2_, dn_2,3_) with dm_1,2_, dm_2,3_, dm_3,4_.

When a set of values matches identically in sequence, the value of (1 + *η*) is 3. Correlate the position information of P_n_^3^ with that of Q_M_^1^ to determine the core’s original formation orientation.

In this figure, the radial axis represents resistivity values in Ω·m, while 0°, 30°,..., 330° indicate different azimuthal angles on the core sample. The percentages on the right side represent water saturation values.

Handling Ambiguity: In cases where applying the above method results in multiple possible correspondences for the core’s in-situ position (e.g., if dn_1+η,2+η_, and dn_γ+η, γ+1+η_ both equal their corresponding dm values, making both P_N_^1^ and P_N_^γ^ appear to correspond with Q_M_^1^), the positional correspondence of the minimum resistivity values can be jointly utilized. Specifically, correlate the positions of the minimum resistivity value from the core measurements with the position of the minimum resistivity value from the resistivity image logging data to jointly determine the core’s precise original azimuthal orientation within the formation.

The successful execution of this work requires permission from the oilfield company. Typically, obtaining electrical imaging data requires authorization from the company, and conducting experiments with the cores also necessitates approval from the department responsible for core management.

The protocol described in this peer-reviewed article is published on protocols.io (https://dx.doi.org/10.17504/protocols.io.kqdg31k77l25/v1) and is included for printing purposes as S1 File. Informed consent was obtained from all human participants involved in this study. The authors declare no competing interests. All data generated or analyzed during this study are available in the protocols.io repository and can be obtained from the corresponding author upon reasonable request.

## Expected results

**Fig 3:**
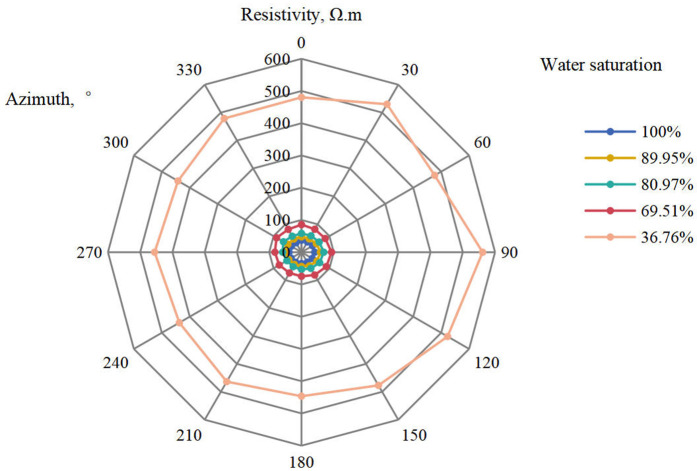
Resistivity distribution of sample 159 from well ST12. From the test data, it can be observed that at different water saturation levels, the resistivity of the rock core still exhibits some variation along different directions.

Taking Well ST12 as an example, Sample 159 was acquired at a depth of 7076 meters. With the formation water saturation at 32.8%, after restoring the core to this fluid saturation condition, the cylindrical core radial resistivity measurement method was used to obtain resistivity data in different directions for this sample, as shown in Fig 3.

The specific procedure is described as follows:

Electrical imaging logs acquire a vast volume of raw data, with approximately over 1,000 data points per meter. The final log is generated by integrating measurements from each individual electrode. As this study focuses solely on the issue of core orientation, the principles underlying electrical imaging logging are not within the scope of this research. Only the processed images from the electrical imaging log are utilized for interpretation purposes. Primary reference is made to the FMI-DYN plot on the right. In this plot, color intensity represents the relative level of conductivity values. The ratio of the color scale values for any two arbitrarily chosen points can be considered as the relative ratio of their conductivity values. The previously measured resistivity values are converted into conductivity values (since conductivity is the reciprocal of resistivity). On the plot, areas with higher conductivity appear darker, while areas with lower conductivity appear lighter.

**Fig 4:**
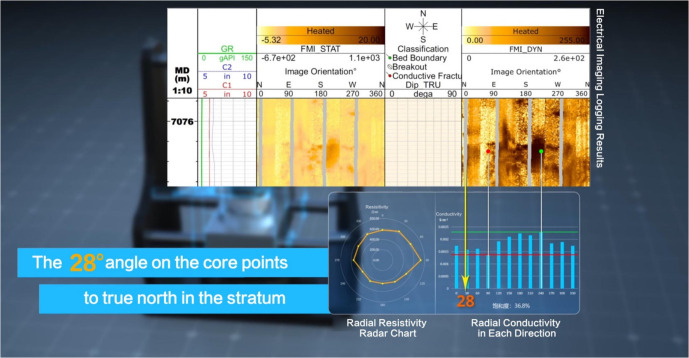
Directional conductivity of core sample 159 from well ST12 correlated with electrical imaging. This figure illustrates the process of comparing the radial resistivity test values of a cylindrical rock core with electrical imaging logging data to determine the original orientation of the core in the formation.

**Step 1:** On the ST12 image log, select the locations with the darkest and lightest colors. Taking Fig 4 as an example, the darkest color is observed at 212°, and the lightest color is observed at 62°.

**Step 2:** Record the original azimuth (orientation in the formation) of these points. The Hue, Saturation, and Brightness (HSB values) of the color at each point are read and recorded as shown in Table 1. (Using Windows’ built-in “Paint” tool, you can achieve this operation. First, use the “Color Picker” to select the area you want to test, then click on “Edit Colors” to view the hue, saturation, and brightness values).

**Table 1:** Comparison of different core orientation techniques.

Comparison Metric	Directional Coring [42,43]	Image Matching [43–45]	Paleomagnetic Analysis [46]	Resistivity-based Core Orientation
Need Pre-job Design	Yes	No	No	No
Need Special Process	Yes	Yes	Yes	No
Operational Cost	Special drilling rigs and tools, and a higher risk	High costs for software and computing resources	Specialized laboratory environments, and significant time cost	Low
Applicable Samples	Cylindrical	Cylindrical	Cuboid (requires cutting)	Cylindrical

**Step 3:** Conductivity is the reciprocal of resistivity. A comparison with core conductivity measurements taken in different directions showed that the core’s conductivity was maximum at 212° and minimum at 62°. Cross-referencing with the ST12 micro-resistivity image log data confirmed that the relative conductivity values at these two orientations are consistent.

**Fig 5:**
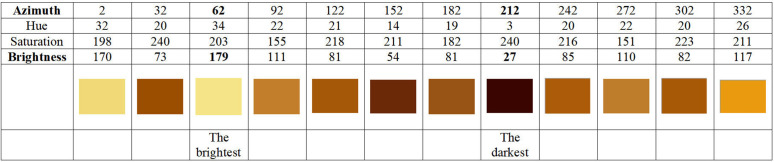
Original formation orientation readings mapped on the electrical imaging log. This figure demonstrates how the brightest and darkest values are obtained by extracting data from point imaging logging images.

**Step 4:** As shown in Fig 5, the brightness at 212° in the resistivity imaging log is only 27. Under conditions closest to the formation fluid saturation, the minimum resistivity measured on the cylindrical core is 437.23 Ω.m. This resistivity minimum corresponds to a marked azimuth of 240° on the core. The difference between the marked azimuth on the full-diameter core and the azimuth in the electrical imaging log indicates that the direction 28° from the marked zero reference line on the core points toward true north in the formation. The radial resistivity test was conducted with a 30° interval between adjacent azimuths. Therefore, more precisely, the orientation 28° ± 15° from the marked zero line on the core points toward true north in the original formation.

If higher accuracy is required, the radial resistivity test can be performed with denser angular sampling around the direction to be determined, measuring resistivity values in more closely spaced orientations.

## Discussion

Compared with the previously mentioned methods—directional coring, image matching, and paleomagnetic analysis—this method offers distinct advantages, as presented in Table 1.

The resistivity-based core orientation method introduced in this paper primarily involves liquid pressurization and saturation, a process that takes about 4 hours or more. Measuring resistivity under varying saturation states can typically be completed within 8 hours. Under normal working conditions, each operator can complete resistivity-based core orientation for 150 samples per year, improving work efficiency by 3 to 3.75 times. Moreover, this method preserves the cylindrical form of the core intact. This ensures no physical loss of these valuable geological materials after testing and leaves the basic properties of the samples unaffected.

However, it should be noted that this method still has certain limitations in practical application:

1. It must rely on electrical imaging logging having been conducted in the well. If this test was not planned and acquired in the field, the method becomes inapplicable.
2. The method requires the target sample to exhibit representative anisotropy. Its accuracy increases with stronger reservoir anisotropy. Conversely, if the reservoir rock is isotropic, the method fails to achieve the expected results. In such cases, core orientation is typically deemed unnecessary, as physical properties are similar in all directions.

In conclusion, the resistivity-based core orientation technique is feasible for reservoirs exhibiting anisotropy and offers broad applicability. Compared to established methods—directional coring, image matching, and paleomagnetic analysis—it holds distinct advantages in both cost-effectiveness and practicality, meriting broader application.

## Conclusions

Cores are typically acquired as cylindrical samples through drilling. Therefore, orientation techniques that work directly on cylindrical samples have broader applicability than methods like paleomagnetic analysis, which require cutting cores into cuboids. To address this challenge, we developed a method correlating radial resistivity measurements of cylindrical cores with electrical imaging logging data, establishing the resistivitybased core orientation technique. This approach features user-friendly procedures and low operational costs. It effectively delineates the original formation orientation in reservoirs by leveraging the resistivity anisotropy characteristics of rocks. Due to its cost-effectiveness and operational simplicity, resistivity-based core orientation will enable directional restoration for a greater volume of cores. This technique provides critical technical support for determining paleocurrent directions and studying depositional environments, while also establishing a robust foundation for engineering geological design.

## Supporting information

S1 File Contains the operating procedures for resistivity-based core orientation. (PDF)

S2 File Gives the explanation of the minimal data set definition. (DOCX)

S3 File Includes the raw data and calculation process for whole-diameter core testing using the radial resistivity method. (XLSX)

S4 File Provides the electrical imaging logging data for ST12. (PDF)

## References

[1] He JH, Li M, Zhou KM, Zeng L, Li N, Yang Y, et al. Radial resistivity measurement method for cylindrical core samples. Interpretation. 2020;8(4):T1071–80. doi: 10.1190/int-2019-0213.1

[2] Qi BQ, He HJ, He L, Miao Q, Zhao N, Huang H, et al. Rock electric analysis and gas zone identification of karst reservoirs, north slope of central Sichuan paleouplift. Nat Gas Explor Dev. 2024;47(1):24–32. doi: 10.12055/gaskk.issn.1673-3177.2024.01.003

[3] Gladchenko ES, Gubanova AE, Orlov DM, Koroteev DA. Kriging-boosted CR modeling for prompt infill drilling optimization. Petroleum. 2024;10(1):39–48. doi: 10.1016/j.petlm.2023.09.003

[4] Tjia HD. Sea-level changes in the tectonically stable Malay-Thai Peninsula. Quat Int. 1996;31:95–101. doi: 10.1016/1040-6182(95)00025-e

[5] Chen M, Yan M, Kang Y, Cao W, Bai J, Li P. Stress sensitivity of multiscale pore structure of shale gas reservoir under fracturing fluid imbibition. Capillarity. 2023;8(1):11–22. doi: 10.46690/capi.2023.07.02

[6] Wang K, Zhang G, Du F, Wang Y, Yi L, Zhang J. Simulation of directional propagation of hydraulic fractures induced by slotting based on discrete element method. Petroleum. 2023;9(4):592–606. doi: 10.1016/j.petlm.2022.04.007

[7] He JH, Dang LR, Wang LJ, Kang Q, Zhang BJ, Zhang C. Exploring the prospects and challenges of petrophysics research from the perspective of materials physics. Adv Geo-Energy Res. 2025;16(2):95–8. doi: 10.46690/ager.2025.05.02

[8] Aadnoy BS. In-situ stress directions from borehole fracture traces. J Pet Sci Eng. 1990;4(2):143–53. doi: 10.1016/0920-4105(90)90022-u

[9] Zhao XG, Wang J, Cai M, Ma LK, Zong ZH, Wang XY, et al. In-situ stress measurements and regional stress field assessment of the Beishan area, China. Eng Geol. 2013;163:26–40. doi: 10.1016/j.enggeo.2013.05.020

[10] Lu M, Su Y, Zhan S, Almrabat A. Modeling for reorientation and potential of enhanced oil recovery in refracturing. Adv Geo-Energ Res. 2020;4(1):20–8. doi: 10.26804/ager.2020.01.03

[11] Brudy M, Zoback MD. Drilling-induced tensile wall-fractures: implications for determination of in-situ stress orientation and magnitude. Int J Rock Mech Mining Sci. 1999;36(2):191–215. doi: 10.1016/s0148-9062(98)00182-x

[12] Li Y, Schmitt DR. Drilling‐induced core fractures and in situ stress. J Geophys Res. 1998;103(B3):5225–39. doi: 10.1029/97jb02333

[13] Lu Y, Jin Y, Li H. Impact of capillary pressure on micro-fracture propagation pressure during hydraulic fracturing in shales: An analytical model. Capillarity. 2023;8(3):45–52. doi: 10.46690/capi.2023.09.01

[14] Cao X, Liu Z, Hu C, Song X, Quaye JA, Lu N. Three-dimensional geological modelling in earth science research: an in-depth review and perspective analysis. Minerals. 2024;14(7):686. doi: 10.3390/min14070686

[15] Pryazhnikov MI, Zhigarev VA, Minakov AV, Nemtsev IV. Spontaneous imbibition experiments for enhanced oil recovery with silica nanosols. Capillarity. 2023;10(3):73–86. doi: 10.46690/capi.2024.03.02

[16] Rogers SF, Bailey DE, Kingdon A. Orientation of drill core by use of borehole geophysical imaging. Appl Earth Sci. 2000;109(3):184–90. doi: 10.1179/aes.2000.109.3.184

[17] Azimian A. A new method for improving the RQD determination of rock core in borehole. Rock Mech Rock Eng. 2015;49(4):1559–66. doi: 10.1007/s00603-015-0789-8

[18] Call RD, Savely JP, Pakalnis R. A simple core orientation technique. Proceeding of the Third International Conference on Stability in Surface Mining. Vancouver, Society of Mining Engineers of AIME, New York. 1982. pp. 465–81.https://www.cnitucson.com/publications/1982_Simple-Cor-Orient-Tech-Call-Savely-Pakalnis.pdf

[19] Nasab SK, Assadipour M, Maghami M. Application of Van Ruth Wire Line Core Orientator at The Sarcheshmeh Open Pit Mine. l International Mining Congress and Exhibition of Turkey-IMCET, 2003. https://api.maden.org.tr/uploads/portal/resimler/ekler/e04e05fbe48920b_ek.pdf

[20] Hailwood EA, Ding F. Palaeomagnetic reorientation of cores and the magnetic fabric of hydrocarbon reservoir sands. Geol Soc London Special Publications. 1995;98(1):245–58. doi: 10.1144/gsl.sp.1995.098.01.15

[21] Cohen A, Campisano C, Arrowsmith R, Asrat A, Behrensmeyer AK, Deino A, et al. The Hominin Sites and Paleolakes Drilling Project: inferring the environmental context of human evolution from eastern African rift lake deposits. Sci Dril. 2016;21:1–16. doi: 10.5194/sd-21-1-2016

[22] Chen DY. Directionally Coring Technique. Nat Gas Ind. 1990;10(8):43–4. doi: CNKI:SUN:TRQG.0.1990-06-010

[23] Li B. Application of oriented coring technology in Qinghai oilfield. Nat Gas Ind. 1999;19(6):91–2. doi: CNKI:SUN:TRQG.0.1999-06-027

[24] Feng YG. Diamond core directional drilling system. Equip Geotech Eng. 2006;7(5):16–8. doi: 10.3969/j.issn.1009-282X.2006.05.003

[25] Zhu HY, Cai ZS, Wang Q, Cheng HW, Zhang Z. Research and application of deep drilling technology methods. Eng Geol. 2013;14(6):26–31. doi: 10.3969/j.issn.1009-282X.2013.06.005

[26] Zabidi H, De Freitas MH. Re-evaluation of rock core logging for the prediction of preferred orientations of karst in the Kuala Lumpur Limestone Formation. Eng Geol. 2011;117(3–4):159–69. doi: 10.1016/j.enggeo.2010.10.006

[27] Zhang JF, Ma XG, Wang ZL, Gao JH. Application of fidelity imaging to the Paleogene near-source fans, Lvda X structural area in the Bohai Sea. Nat Gas Explor Dev. 2023;46(1):57–64. doi: 10.12055/gaskk.issn.1673-3177.2023.01.007

[28] Shan L, Liu Y, Du K, Paul S, Zhang X, Hei X. Drilling rock image segmentation and analysis using segment anything model. Adv Geo-Energy Res. 2024;12(2):89–101. doi: 10.46690/ager.2024.05.02

[29] Ureel S, Momayez M, Oberling Z. Rock core orientation for mapping discontinuities and slope stability analysis. Int J Res Eng Technol. 2013;2(7):1–8. https://www.cnitucson.com/publications/2013_Ureel_IJRET20130207001.pdf

[30] Akmal L, Pyrcz MJ. Physics-based discrepancy modeling for well log imputation. Math Geosci. 2025;57(7):1235–64. doi: 10.1007/s11004-025-10203-7

[31] Grimaud J-L, Desassis N, Chourio-Camacho D, Renard D, Pereira M, Ors F, et al. Kriging alluvial thicknesses in valley bottoms using nonstationary geometric anisotropies. Math Geosci. 2025;57(7):1379–99. doi: 10.1007/s11004-025-10200-w

[32] Nie X, Zou CC, Xiao K, Xu J, Niu YX, Kong GS. Core spatial position of WFSD-1 borehole with borehole imaging logging data. Progr Geophys. 2012;27:0075–82. doi: 10.6038/j.issn.1004-2903.2012.01.009

[33] Nie X, Zou C, Pan L, Huang Z, Liu D. Fracture analysis and determination of in-situ stress direction from resistivity and acoustic image logs and core data in the Wenchuan Earthquake Fault Scientific Drilling Borehole-2 (50–1370 m). Tectonophysics. 2013;593:161–71. doi: 10.1016/j.tecto.2013.03.005

[34] Mullender TAT, Velzen AJ, Dekkers MJ. Continuous drift correction and separate identification of ferrimagnetic and paramagnetic contributions in thermomagnetic runs. Geophys J Int. 1993;114(3):663–72. doi: 10.1111/j.1365-246x.1993.tb06995.x

[35] Dunlop DJ. Theory and application of the Day plot (Mrs/Ms versus Hcr/Hc) 1. Theoretical curves and tests using titanomagnetite data. J Geophys Res. 2002;107(B3). doi: 10.1029/2001jb000486

[36] Nie X, Song J, Wei W, Cai J. Digital rock physics and resistivity well logging interpretation in unconventional reservoirs: Advances and prospects. Adv Geo-Energy Res. 2025;18(3):287–90. doi: 10.46690/ager.2025.12.07

[37] Akingboye AS, Bery AA. Rock mass quality evaluation via statistically optimized geophysical datasets. Bull Eng Geol Environ. 2023;82(10). doi: 10.1007/s10064-023-03380-4

[38] Akingboye AS. RQD modeling using statistical-assisted SRT with compensated ERT methods: Correlations between borehole-based and SRT-based RMQ models. Phys Chem Earth Parts A/B/C. 2023;131:103421. doi: 10.1016/j.pce.2023.103421

[39] Akingboye AS. Electrical and seismic refraction methods: fundamental concepts, current trends, and emerging machine learning prospects. Discov Geosci. 2025;3(1). doi: 10.1007/s44288-025-00169-8

[40] Sier MJ, Langereis CG, Dupont-Nivet G, Feibel CS, Joordens JCA, van der Lubbe JHJL, et al. The top of the Olduvai Subchron in a high-resolution magnetostratigraphy from the West Turkana core WTK13, hominin sites and Paleolakes Drilling Project (HSPDP). Quat Geochronol. 2017;42:117–29. doi: 10.1016/j.quageo.2017.08.004

[41] He JH, Tang ZJ, Zou MW. New technology for radial resistivity measurement of rock core. Nat Gas Ind. 2022;42(9):75. doi: 10.3787/j.issn.1000-0976.2022.09.008

[42] Wang Y, Li X, Ma Y, Shi S, Zhou Q, Chou J, et al. Design and experiments of directional core drilling tool. Applied Sciences. 2025;15(21):11612. doi: 10.3390/app152111612

[43] Xia H, Jiang S. Geostress effect on resistivity and its relevant correction method. Petroleum. 2023;9(3):412–8. doi: 10.1016/j.petlm.2021.06.003

[44] Xue T, Liu Y, Yang X, Liu J. A study on the features of fractures in the volcanic reservoirs of Shengping gas field. Nat Gas Ind. 2009;29(8):35–7. doi: 10.3787/j.issn.1000-0976.2009.08.011

[45] Zhang S, Peng C, Zou C. An automatic method for core orientation based on planar geologic features in drill-core scans and microresistivity images. IEEE Access. 2022;10:116004–13. doi: 10.1109/access.2022.3214197

[46] William EH. Paleomagnetic methods for orientation of borehole cores, horizon cleveland field, Ochiltree County, Texas: ABSTRACT. Bulletin. 1982;66. doi: 10.1306/03b59a9e-16d1-11d7-8645000102c1865d

