# Supplementary material for: Resistivity method-based rock core orientation experimental protocol
Source: PLoS One. 2026 Mar 2;21(3):e0342912. doi: 10.1371/journal.pone.0342912 (PMC12952577; doi:10.1371/journal.pone.0342912)
Supplement: S1 File — (PDF) [file pone.0342912.s001.pdf]

Oct 05, 2025

Rock Core Orientation Technology Using Resistivity Method

DOI
dx.doi.org/10.17504/protocols.io.kqdg31k77l25/v1

J.H[1], Z.T.[1], J.W.[1], L.W.[1], H.Y.[1], Q.Z.[1], X.T.[1], Q.K.[1], H.C.[1], L.Z.[1], W.L.[1]

[1]Exploration and Development Research Institute, Southwest Oil and Gas Field Company, PetroChina

HeJiahuan

Anonymous

OPEN ACCESS

DOI: **https://dx.doi.org/10.17504/protocols.io.kqdg31k77l25/v1**

Protocol Citation: J.H, Z.T., J.W., L.W., H.Y., Q.Z., X.T., Q.K., H.C., L.Z., W.L. 2025. Rock Core Orientation Technology Using Resistivity Method. **protocols.io** **https://dx.doi.org/10.17504/protocols.io.kqdg31k77l25/v1**

License: This is an open access protocol distributed under the terms of the **Creative Commons Attribution License**, which permits unrestricted use, distribution, and reproduction in any medium, provided the original author and source are credited

Protocol status: Working
We use this protocol and it's working

Created: September 07, 2025

Last Modified: October 05, 2025

Protocol Integer ID: 226668

Keywords: rock core orientation technology, original orientation of core sample, based core orientation technique, core orientation technique, core orientation, crucial for geological engineer, paleomagnetic testing, geological insight, downhole electrical imaging, core sample, images of the core, conventional drilling, geological engineer, subsurface rock, core into cube, cutting core, physical properties of subsurface rock, available cylindrical core, resistivity method, most core, widespread use on most core, orientation, electrical resistivity, using resistivity method, core, original orientation, such as hydraulic fracturing design, resistivity, hydraulic fracturing design

Disclaimer

Competing interests: All authors declare that they have no conflicts of interest.

Abstract

Restoring the original orientation of core samples in the formation is crucial for geological engineers to better understand the physical properties of subsurface rocks, holding significant importance for engineering operations such as hydraulic fracturing design. However, over 99% of cores worldwide are acquired via conventional drilling coring, making it difficult to determine their orientation during the trip out of the hole. While directional coring and cutting core into cubes for paleomagnetic testing aim to achieve core orientation, the complexity of these processes prevents their widespread use on most cores. To address this, this paper develops an electrical resistivity-based core orientation technique suitable for the more universally available cylindrical cores. It obtains resistivity scanning images of the core, which are then meticulously correlated with downhole electrical imaging logging results. This enables core orientation, providing exploration professionals with the means to gain geological insights incorporating directional information.

Image Attribution

Figure 1. Resistivity Distribution of Sample 159 from Well ST12. Processed electrical imaging log used (referred to as the FMI-DYN plot on the right); color intensity on the plot represents relative conductivity values. The log is generated by integrating measurements from each individual electrode; raw imaging data volume is approximately over 1,000 data points per meter.

Figure 2. Directional Conductivity of Core Sample 159 from Well ST12 Correlated with Electrical Imaging

Table 1. Original formation orientation readings mapped on the electrical imaging log.

Overlay text visible in Figure 2: "The 28° normal direction of the core points to true north in the original formation."

Guidelines

Materials and Methods (procedural / methodology content present on these pages):

- To achieve in-situ parameter measurements on core samples, first determine the fluid saturation corresponding to the specific well depth. For high-maturity gas reservoirs, the water saturation at the target depth can be directly referenced. A full-diameter core sample is then fully saturated with formation water. A gas-displacing-water process is employed to establish saturation conditions consistent with those in the formation. Simultaneously, the cylindrical sample radial resistivity measurement method is used to test the resistivity values in different directions.
- The radial resistivity formula for rock core is given as follows (equation as shown on page):

  $\rho = (K / K') L R$ (Equation 1)

  where ρ denotes resistivity, in Ω·m;

  $K(\alpha)$ is defined by an integral expression shown on the page (K' denotes the derivative of K);

  2α denotes the supplementary angle to the central angle of the curved electrode sheet contacting the core;

  L denotes the core length, in m;

  R denotes the apparent resistance value, in Ω.

Taking the 159 sample of ST12 as an example, the workflow of core orientation using the resistivity method is described as follows:

Materials

Items and materials explicitly mentioned on these pages:

- Full-diameter cylindrical core samples
- Formation water
- Gas (for gas-displacing water process)
- Equipment/method for radial resistivity measurement of cylindrical samples (radial resistivity measurement method)
- Downhole electrical imaging logging (used for correlation with core resistivity scans)
- Curved electrode sheet contacting the core (part of the radial resistivity measurement apparatus)
- Apparent resistance measurement capability/instrument to obtain R (apparent resistance, in Ω)
- Means to mark/divide the core circumference into N equal segments and a reference point "O" (for segment-based measurements)
- Tools to measure core length (L, in m)
- Electrical imaging logging tool and associated individual electrodes (raw data acquisition; ~1,000+ data points per meter)
- Data processing/software to generate FMI-DYN (or equivalent) plots and to convert resistivity values to conductivity values for imaging interpretation

Troubleshooting

Before start

Preparatory notes found on these pages:
- Determine fluid saturation corresponding to the specific well depth before measurements.
- For high-maturity gas reservoirs, reference the water saturation at the target depth.
- Ensure core saturation conditions replicate formation conditions (saturate with formation water and use gas-displacing-water process to reach desired saturation).
- Prepare to perform radial resistivity measurements and to divide/mark the core circumference into N equal segments with a reference point "O".

Materials and Methods

1 After fully saturating the full-diameter sample with water, different water saturation states of the full-diameter core are achieved through gas displacing water. Using the radial resistivity measurement method (with the curved electrode pieces of the testing device set at a 90° angle; for the testing principle, refer to He JH, Li M, Zhou KM, Zeng L, Li N, Yang Y, Xiao D, Huang M. Radial resistivity measurement method for cylindrical core samples. Interpretation. 2020 Nov;8(4), T1071–T1080. https://doi.org/10.1190/INT-2019-0213.1), the resistivity values in various directions are measured under different water saturation conditions. In this example, the resistivity is measured every 30°. The core is divided into 12 equal segments at 30° intervals, labeled P_1, P_2, ..., P_{12}. The measured resistivity values are provided in S2.

2 Based on the calculated resistivity values, the conductivity values in each direction are computed using the reciprocal relationship between conductivity and resistivity. Specific calculated values can be found in Table S2 in the attachment.

3 According to the water saturation data from the corresponding well depth provided by the field, the conductivity values converted from the resistivity measured under the closest water saturation condition in the radial resistivity test are selected and compared with the conductivity values obtained from the electrical imaging logging.

4 Based on the radial resistivity measurement results, it is straightforward to identify the rotation angles θ_{max} and θ_{min} corresponding to the maximum resistivity value R_{max} and the minimum resistivity value R_{min} respectively, and mark these orientations on the core. It is noted that among the conductivity values measured every 30°, the resistivity values at 90° and 240° are the maximum (561.43 Ω·m) and minimum (437.23 Ω·m), respectively. We denote 90° as the angle θ_{max} with the maximum resistivity, and 240° as the angle θ_{min} with the minimum resistivity. In the core test results, the point with the minimum conductivity is P_4, and the point with the maximum conductivity is P_9.

5 A comparison is made with the conductivity values from the electrical imaging log. At the corresponding depth of the ST12 well in the electrical imaging log, the brightest and darkest values are identified, which are represented using conductivity values.Record the original azimuth (orientation in the formation) of these points. The Hue, Saturation, and Brightness (HSB values) of the color at each point are read and recorded as shown in Table 1. （Using Windows' built-in "Paint" tool, you can achieve this operation. First, use the "Color Picker" to select the area you want to test, then click on "Edit Colors" to view the hue, saturation, and brightness values.）

	**Azimuth**	**2**	**32**	**62**	**92**	**122**	**152**	**182**	**212**	**242**	**272**	**302**	**332**
	Hue	32	20	**34**	22	21	14	19	3	20	22	20	26
	Saturation	198	240	**203**	155	218	211	182	240	216	151	223	211
	**Brightness**	**170**	**73**	**179**	**111**	**81**	**54**	**81**	**27**	**85**	**110**	**82**	**117**
				The brightest					The darkest				

On the corresponding electrical imaging log, the darkest point is observed at 212°, representing the location with the lowest resistivity. The electrical imaging log is then divided into 12 equal segments, and the corresponding values are read from each segment. It can be observed that among these 12 points, the position with the darkest color and the lowest resistivity is at 212°, with a brightness value of 27, while the brightest position with the highest resistivity is at 62°, with a brightness value of 179.

6 Since the colors in the electrical imaging log reflect the relative magnitude of conductivity, identifying the darkest and brightest points at the corresponding depth is particularly important. Based on the above, the measurement points can be labeled as Q_1, Q_2, Q_3, ..., Q_{12} at 2°, 32°, 62°, etc., respectively. The position with the darkest color and the lowest resistivity is at 212°, recorded as Q_8, while the brightest position with the highest resistivity is at 62°, with a brightness value of 179, recorded as Q3.

7 By comparing the relative magnitudes of radial conductivity and electrical imaging logging, it can be observed that P_9 corresponds to the same location as Q_8, and P_4 corresponds to Q_3.

8 P_1 represents the starting point at 0° on the core. By comparing the position of P_1 in the electrical imaging log image, it can be determined that the 28° angle on the core aligns with true north direction in the formation.

Protocol references

1. He JH, Li M, Zhou KM, Zeng L, Li N, Yang Y, Xiao D, Huang M. Radial resistivity measurement method for cylindrical core samples. Interpretation. 2020 Nov;8(4), T1071-1080. https://doi.org/10.1190/INT-2019-0213.1

2. Qi BQ, He HJ, He L, Miao Q, Zhao N, Huang H, Lu SH. Rock electric analysis and gas zone identification of karst reservoirs, north slope of central Sichuan paleouplift. Natural Gas Exploration and Development. 2024 Feb;47(1): 24-32. http://dx.doi.org/10.12055/gaskk.issn.1673-3177.2024.01.003

3. Elivazeta SG, Anna EG, Denis MO, Dmitry AK. Kriging-boosted CR modeling for prompt infill drilling optimization. Petroleum. 2024 March;10(1): 39-48. https://doi.org/10.1016/j.petlm.2023.09.003

4. Tija HD. Sea level changes in the technically stable Mala-Thai Peninsula. Quaternary International. 1996 Dec;31:95-101. https://dx.doi.org/10.1016/1040-6182(95)00025-E

5. Chen MJ, Yan ML, Kang YL, Cao W, Bai JJ, Li PS. Stress sensitivity of multiscale pore structure of shale gas reservoir under fracturing fluid imbibition. Capillarity. 2023 Jun;8(1): 11-22. https://doi.org/10.46690/capi.2023.07.02

6. Wang K, Zhang GD, Du F, Wang YH, Yi LP, Zhang JQ. Simulation of directional propagation of hydraulic fractures induced by slotting based on discrete element method. Petroleum. 2023 Dec;04: 592-606. https://www.sciencedirect.com/science/article/pii/S2405566122000438

7. He JH, Dang LR, Wang LJ, Kang Q, Zhang BJ, Zhang C. Exploring the prospects and challenges of petrophysics research from the perspective of materials physics. Advances in Geo-Energy Research. 2025 Jan;16(2): 95-98. https://doi.org/10.46690/ager.2025.05.02

8. Aadnoy BS. In-situ stress directions from borehole fracture traces. Journal of Petroleum Science and Engineering. 1990 May;4(2), 143–153. https://doi.org/10.1016/0920-4105(90)90022-u

9. Zhao XG, Wang J, Cai M, Ma LK, Zong XY, Su R, Chen WM, Zhao HG, Chen QC, An QM, Qin XH, Ou MY, Zhao JS. In-situ stress measurements and regional stress field assessment of the Beishan area, China. Engineering Geology. 2013 Aug;163: 26-40. http://dx.doi.org/10.1016/j.enggeo.2013.05.020

10. Lu MJ, Su YL, Zhan SY, Abduhadi A. Modeling for reorientation and potential of enhanced oil recovery in refracturing. Advances in Geo-Energy Research. 2020 Feb;4(1): 20-28. https://doi.org/10.26804/ager.2020.01.03

11. Brudy M, Zoback MD. Drilling-induced tensile wall-fractures: implications for determination of in-situ stress orientation and magnitude. International Journal of Rock Mechanics and Mining Sciences. 1999 Feb;36: 191-215. https://doi.org/10.1016/S0148-9062(98)00182-X

12. Li YY, Douglas RS. Drilling-induced core fractures and in situ stress. Journal of Geophysical Research. 1998 Mar;103(B3), 5225-5239. https://doi.org/10.1029/97JB02333

13. Lu YH, Jin Y, Li HD. Impact of capillary pressure on micro-fracture propagation pressure during hydraulic fracturing in shales: An analytical model. Capillarity. 2023 Aug;8(3): 45-52. https://doi.org/10.46690/capi.2023.09.01

14. Cao XQ, Liu ZM, Hu CL, Song XL, Jonathan AQ, Lu N. Three-Dimensional Geological Modelling in Earth Science Research: An In-Depth Review and Perspective Analysis. Minerals. 2024 Jun;14(7): 486. https://doi.org/10.3390/min14070686

15. Maxim IP, Vladimir AZ, Andrey VM, Ivan VN. Spontaneous imbibition experiments for enhanced oil recovery with silica nanosols. Capillarity. 2024 Dec;10(3): 73-86. https://doi.org/10.46690/capi.2024.03.02

16. Rogers SF, Bailey DE, Kingdon A. Orientation of drill core by use of borehole geophysical imaging. Applied Earth Science. 2000 Dec;193(3): 184-190. https://dx.doi.org/10.1179/aes.2000.109.3.184

17. Abdolaizm AA. New Method for Improving the RQD Determination of Rock Core in Borehole. Rock Mech Rock Eng. 2016 Jul;49: 1559-1566. https://doi.org/10.1007/s00603-015-0789-8

18. Call RD, Savely JP, Pakalnis R. A simple core orientation technique. Proceeding of the Third International Conference on Stability in Surface Mining. Vancouver, Society of Mining Engineers of AIME, New York. 1982:465-481. https://www.cnitucson.com/publications/1982_Simple-Core-Orient-Tech-Call-Savely-Pakalnis.pdf

19. Nasab SK, Assadipour M, Maghami M. Application of Van Ruth Wire Line Core Orientator at The Sarcheshmeh Open Pit Mine. I International Mining Congress and Exhibition of Turkey-IMCET, 2003. https://api.maden.org.tr/uploads/portal/resimler/ekler/e04e05fbe48920b_ek.pdf

20. Hailwood EA, Ding F. Palaeomagnetic reorientation of cores and the magnetic fabric of hydrocarbon reservoir sands. Geological Society London Special Publications. 1995;98, 245-258. http://dx.doi.org/10.1144/GSL.SP.1995.098.01.15

21. Cohen A, Campisano CC, Arrowsmith R, Asrat A, Behrensmeyer AK, Deino A, Feibel C, Hill A, Johnson R, Kingston J, Lamb H, Lowenstein T, Noren A, Olago D, Owen RB, Potts R, Reed K, Renault R, Schäbitz F, Tierecelin JJ, Trauth MH, Wynn J, Ivory S, Brady K, O'Grady R, Rodysill J, Githiri J, Russell J, Foerster V, Dommain R, Rucina S, Deocampo D, Russell J, Billingsley A, Beck C, Dorenbeck G, Dullo L, Feary D, Garello D, Gromig R, Johnson T, Junginger A, Karanja M, Kimburi E, Mbuthia A, McCartney T, McNulty E, Muiruri V, Nambiro E, Negash EW, Njagi D, Rabideaux N, Raub T, Sier MJ, Smith P, Urban J, Warren M, Wilson JN, Yadeta M, Yost C, Zinaye B. The Hominin Sites and Paleolakes Drilling Project: inferring the environmental context of human evolution from eastern African rift lake deposits. Scientific Drilling. 2016 Feb;21, 1-16. http://dx.doi.org/10.5194/sd-21-1-2016

22. Chen DY. Directionally Coring Technique. Natural Gas Industry. 1990 Jul;10(8): 43-44.https://doi.org/CNKI:SUN:TRQG.0.1990-06-010

23. Li B. Application of Oriented Coring Technology in Qinghai Oilfield. Naturals Gas Industry. 1999 Nov; 19(6): 91-92. https://doi.org/CNKI:SUN:TRQG.0.1999-06-027

24. Feng YG. Diamond Core Directional Drilling System. Equipment for Geotechnical Engineering. 2006 Oct; 7(5):16-18. http://dx.doi.org/10.3969/j.issn.1009-282X.2006.05.003

25. Zhu HY, Cai ZS, Wang Q, Cheng HW, ZhangZ. Research and Application of Deep Drilling Technology Methods. Engineering Geology. Equipment for Geotechnical Engineering. 2013 Dec;,14(6): 26-31. http://dx.doi.org/10.3969/j.issn.1009-282X.2013.06.005

26. Hareyani Z, Michael HDF. Re-evaluation of rock core logging for the prediction of preferred orientations of karst in the Kuala Lumpur Limestone Formation. Engineering Geology. 2011 Feb; 117: 159-169. https://doi.org/10.1016/enggeo.2010.10.006

27. Zhang JF, Ma XG, Wang ZL, Gao JH. Application of fidelity imaging to the Paleogene near-source fans, Lvda X structural area in the Bohai Sea. Natural Gas Exploration and Development 2023 Mar;46(1): 57-64. http://dx.doi.org/10.12055/gaskk.issn.1673-3177.2023.01.007

28. Shan LQ, Liu YC, Du K, Shovonm P, Zhang XL, Hei XL.Drilling rock image segmentation and analysis using segment anything model. Advances in Geo-Energy Research. 2024 Mar;12(2): 89-101.https://doi.org/10.46690/ager.2024.05.02

29. Ureel S, Momayez M, Oberling Z. Rock core orientation for mapping discontinuities and slope stability analysis. International Journal of Research in Engineering and Technology. 2013 Jul; 2(7): 1-8. https://www.cnitucson.com/publications/2013*Ureel*IJRET20130207001.pdf

30. Laaiba A, Michael JP. Physics-Based Discrepancy Modeling for Well Log Imputation. Mathematical Geosciences. 2025 Jul; 7:1-30. https://doi.org/10.1007/s11004-025-10203-7

31. Jean-Louis G, Nicolas D, Diana C, Didier R, Mike P, Fabien O, Helene T, Paul B, Mark N. Kriging Alluvial Thicknesses in Valley Bottoms Using Nonstationary Geometric Anisotropies. Mathematical Geosciences. 2025 Jul;7:1-21. https://doi.org/10.1007/s11004-025-10200-w

32. Mullender TAT, van Velzen AJ, Dekkers MJ.Continuous drift correction and separate identification of ferrimagnetic and paramagnetic contributions in thermomagnetic runs. Geophysical Journal International. 1993 Sep; 114, 663e672. http://dx.doi.org/10.1111/j.1365-246X.1993.tb06995.x

33. Dunlop DJ. Theory and application of the Day plot (Mrs/Ms versus Hcr/Hc) 2. Application to data for rocks, sediments, and soils. Journal of Geophysical Research. 2002 Mar; 107, 1-15.http://dx.doi.org/10.1029/2001JB000486

34. Mark J S, Cor GL, Guillaume D, Craig SF, Josephine CAJ, Jeroen HJL, Catherine CB, Daniel O, Andrew C, WTK Science team members. The top of the Olduvai Subchron in a high-resolution agnetostratigraphy from the West

Turkana core WTK13, hominin sites and Paleolakes Drilling Project (HSPDP). Quaternary Geochronology. 2017 Oct; 42:117-129. http://dx.doi.org/10.1016/j.quageo.2017.08.004

35. He J H, Tang ZJ, Zou MW. New Technology for Radial Resistivity Measurement of Rock Core. Natural Gas Industry. 2022 Sep; 42(9): 75. https://doi.org/10.3787/j.issn.1000-0976.2022.09.008

36. Xia HQ, Jiang SX. Geostress effect on resistivity and its relevant correction method. Petroleum. 2023 Sep; 9(3): 412-418. https://www.sciencedirect.com/science/article/pii/S2405656121000481

37. Xue T, Liu Y, Yang X, Liu J. A study on the features of fractures in the volcanic reservoirs of Shengping gas field. Natural Gas Industry. 2009 Sep;29(8): 35-37. https://dx.doi.org/10.3787/j.issn.1000-0976.2009.08.011

Acknowledgements

Funding: The authors sincerely thank the CNPC Scientific and Technological Projects(No. 2025D206) and (No. 2025D00108) for their financial support.

The authors also want to thank Ms. Liu Yuanling for the exquisite illustrations she provided for this article.

Data availability: Data are available from the corresponding author upon reasonable request.

Document update: Updated September 7 2025.

Authors' contributions:
Conceptualization, J.H. and Z.T.; methodology, J.H.; software, J.W.; validation, L..W and H.Y.; formal analysis, Q.Z. and X. T.; investigation, Q.K. and H.C.; resources, Q.K. and Z.T.; data curation, H.C.; writing—original draft preparation, J.H.; writing—review and editing, L.Z.; visualization, H.Y.; supervision, H.C.; project administration, W.L.; funding acquisition, Q.K.. All authors have read and agreed to the published version of the manuscript.
