# Supplementary material for: Resistivity method-based rock core orientation experimental protocol
Source: PLoS One. 2026 Mar 2;21(3):e0342912. doi: 10.1371/journal.pone.0342912 (PMC12952577; doi:10.1371/journal.pone.0342912)
Supplement: S2 File — (DOCX) [file pone.0342912.s002.docx]

Figure 1 is derived from calculations based on the data in S3. This Excel file contains the raw experimental test data, including how resistivity is calculated from resistance measurements in different directions. Additionally, the calculation formulas and data charts are preserved in this Excel file, allowing readers to clearly follow the computational process.

The resistivity imaging map in Figure 2 comes from file S4, while the radial conductivity listed below it is converted from the radial resistivity in S3, as conductivity and resistivity have a reciprocal relationship.

The HSB data readings for different directions listed in Table 1 are obtained through the following method: "Record the original azimuth (orientation in the formation) of these points. The Hue, Saturation, and Brightness (HSB values) of the color at each point are read and recorded as shown in Table 1. (Using Windows' built-in 'Paint' tool, you can achieve this operation. First, use the 'Color Picker' to select the area you want to test, then click on 'Edit Colors' to view the hue, saturation, and brightness values.)" The values listed in the table are obtained using the method described above.

In summary, all data presented in this paper have been fully provided in the supplementary files. These data are sufficient to support the reproducibility of the method introduced in this paper. There are no instances of "data not shown" in this work. If reviewers or the editorial board believe that additional data should be disclosed, please specify which data need to be provided in the supplementary materials.
