# Supplementary material for: Resistivity method-based rock core orientation experimental protocol
Source: PLoS One. 2026 Mar 2;21(3):e0342912. doi: 10.1371/journal.pone.0342912 (PMC12952577; doi:10.1371/journal.pone.0342912)
Supplement: S4 File — (PDF) [file pone.0342912.s004.pdf]

Schlumberger *Techlog

FMI* Analysis
Shuangtan12
7055 - 7269m (MD 1/10)

* A Mark of Schlumberger

COMPANY:	[illegible] ny
WELL:	[illegible]
FIELD:	[illegible]
RIG:	Sichuan
COUNTRY:	[illegible]
Date Logg[illegible]:	[illegible]
Surface [illegible]	[illegible]
Elevations:	KB: 0 m DF: [illegible]

FOLD HERE: The well name, location and borehole reference data were furnished by the customer.

Any interpretation, research, analysis, data, results, estimates, or recommendation furnished with the services or otherwise commu[illegible] customer at any time in connection with the services are opinions based on inferences from measurements, empirical relationsh[illegible] inferences, empirical relationships and/or assumptions are not infallible and with respect to which professionals in the industry may [illegible] cannot and does not warrant the accuracy, correctness, or completeness of any such interpretation, research, analysis, data, results, est[illegible] customer acknowledges that it is accepting the services "as is," that Schlumberger makes no representation or warranty, express or im[illegible] respect thereto, and that such services are delivered with the explicit understanding and agreement that any action taken based on th[illegible] own risk and responsibility, and no claim shall be made against Schlumberger as a consequence thereof.

Svc. Order #:	Interpretation Center:	Analyst: W	Process Date:29 May 2018

Remarks:

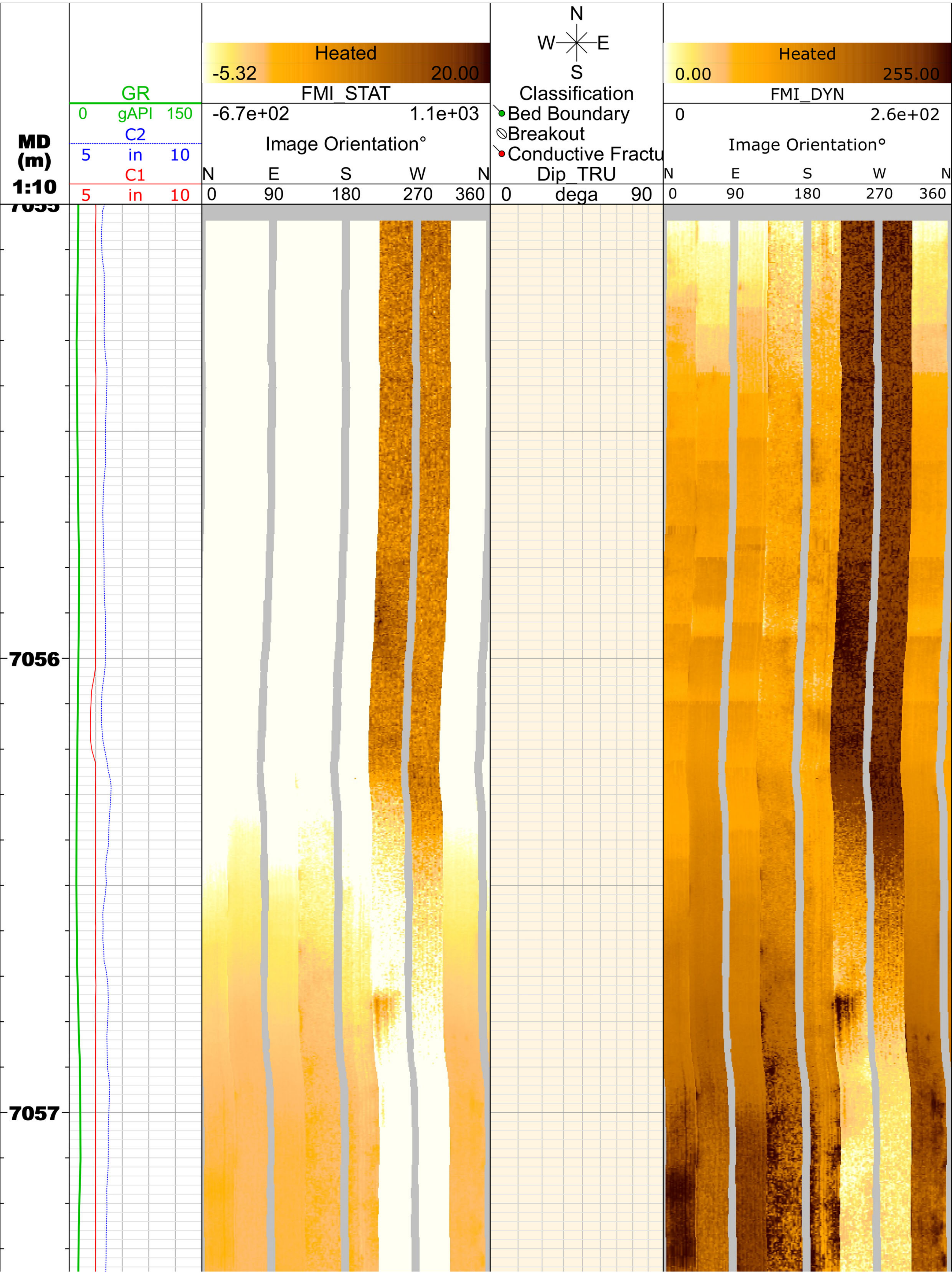
MD
(m)
1:10
GR
0 gAPI 150
C2
5 in 10
C1
5 in 10
Heated
-5.32 20.00
FMI_STAT
-6.7e+02 1.1e+03
Image Orientation°
N E S W N
0 90 180 270 360
N
W E
S
Classification
Bed Boundary
Breakout
Conductive Fractu
Dip_TRU
0 dega 90
Heated
0.00 255.00
FMI_DYN
0 2.6e+02
Image Orientation°
N E S W N
0 90 180 270 360
7056
7057

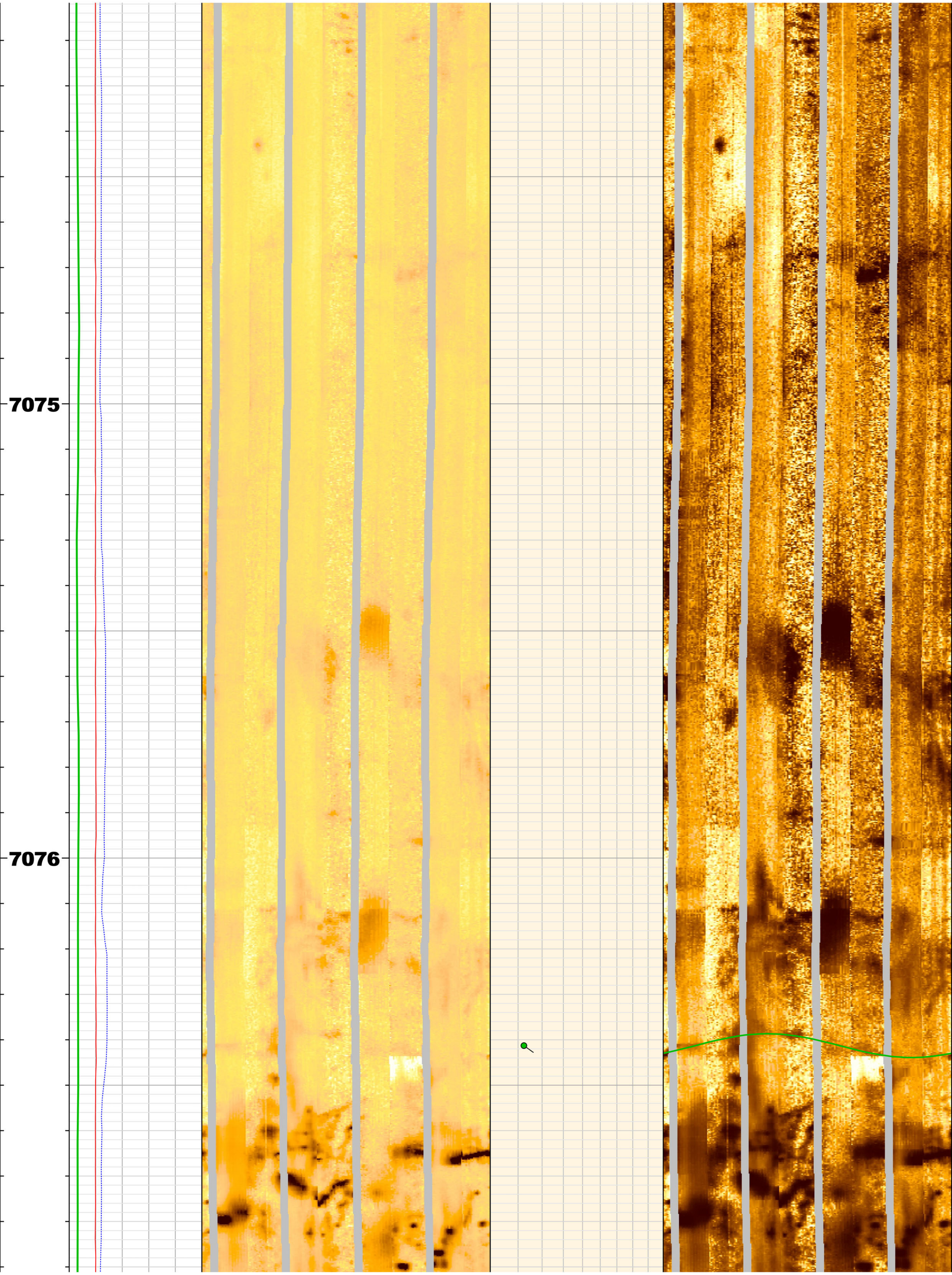
7075
7076
